# Development of genome engineering technologies in cattle: from random to specific

**DOI:** 10.1186/s40104-018-0232-6

**Published:** 2018-01-30

**Authors:** Soo-Young Yum, Ki-Young Youn, Woo-Jae Choi, Goo Jang

**Affiliations:** 1 Department of Veterinary Clinical Science, College of Veterinary Medicine and the Research Institute of Veterinary Science, Seoul National University, Seoul, 08826 Republic of Korea; 2 Farm Animal Clinical Training and Research Center, Institute of GreenBio Science Technology, Seoul National University, PyeongChang-Gun, Gangwon-do 25354 Republic of Korea; 3 Emergence Center for Food-Medicine Personalized Therapy System, Advanced Institutes of Convergence Technology, Seoul National University, SuWon, Gyeonggi-do 16629 Republic of Korea; 4 College of Veterinary Medicine, Seoul National University, #85, Room631, 1 Gwanak-ro, Gwanak-gu, Seoul, 08826 Republic of Korea

**Keywords:** Cattle, CRISPR-Cas9, Genome engineering technologies, Transgenesis, Transposon

## Abstract

The production of transgenic farm animals (e.g., cattle) via genome engineering for the gain or loss of gene functions is an important undertaking. In the initial stages of genome engineering, DNA micro-injection into one-cell stage embryos (zygotes) followed by embryo transfer into a recipient was performed because of the ease of the procedure. However, as this approach resulted in severe mosaicism and has a low efficiency, it is not typically employed in the cattle as priority, unlike in mice. To overcome the above issue with micro-injection in cattle, somatic cell nuclear transfer (SCNT) was introduced and successfully used to produce cloned livestock. The application of SCNT for the production of transgenic livestock represents a significant advancement, but its development speed is relatively slow because of abnormal reprogramming and low gene targeting efficiency. Recent genome editing technologies (e.g., ZFN, TALEN, and CRISPR-Cas9) have been rapidly adapted for applications in cattle and great results have been achieved in several fields such as disease models and bioreactors. In the future, genome engineering technologies will accelerate our understanding of genetic traits in bovine and will be readily adapted for bio-medical applications in cattle.

## Background

Livestock are very important to humans because they provide food resources (meat and/or milk) and other by-products such as leather. Cattle are known as the best animals for producing large amounts of milk and/or meat and are regarded as a valuable protein resource. Additionally, they are utilized for research regarding assisted reproduction technologies such as in vitro fertilization, superovulation, embryo transfer, somatic cell nuclear transfer (SCNT) and cryopreservation, which help us to further our understanding of basic and advanced embryology in animals as well as in humans. Recently, the introduction of new genome technologies such as whole genome sequencing and genome manipulation in cattle, have opened a new era for industrial applications. In this review, we will summarize several genomic engineering technologies for producing genome modified cattle (GMC).

## History of GMC

**Fig. 1:**
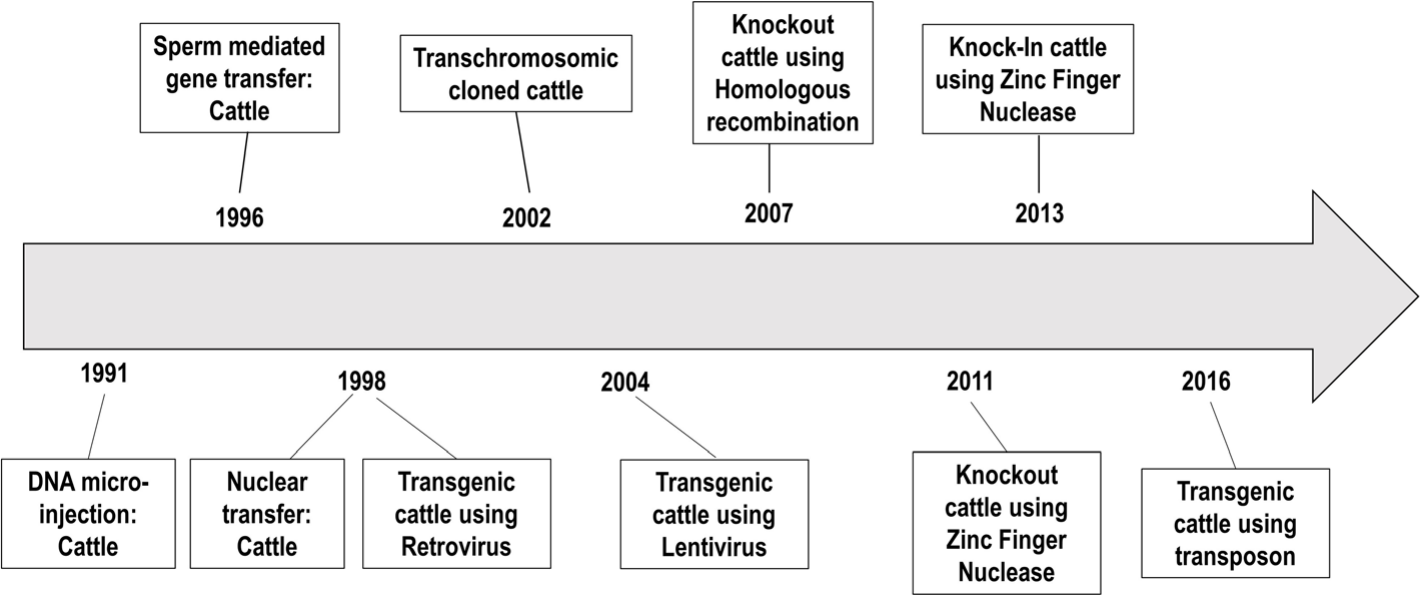
Milestones in the production of transgenic cattle

**Fig. 2:**
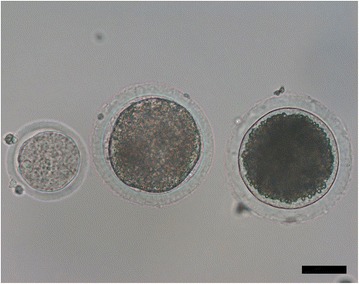
Representative pictures of oocytes. Left: oocyte from rats, Middle: oocyte from cow, Right: oocyte from pigs. Scale = 50 µm

**Fig. 3:**
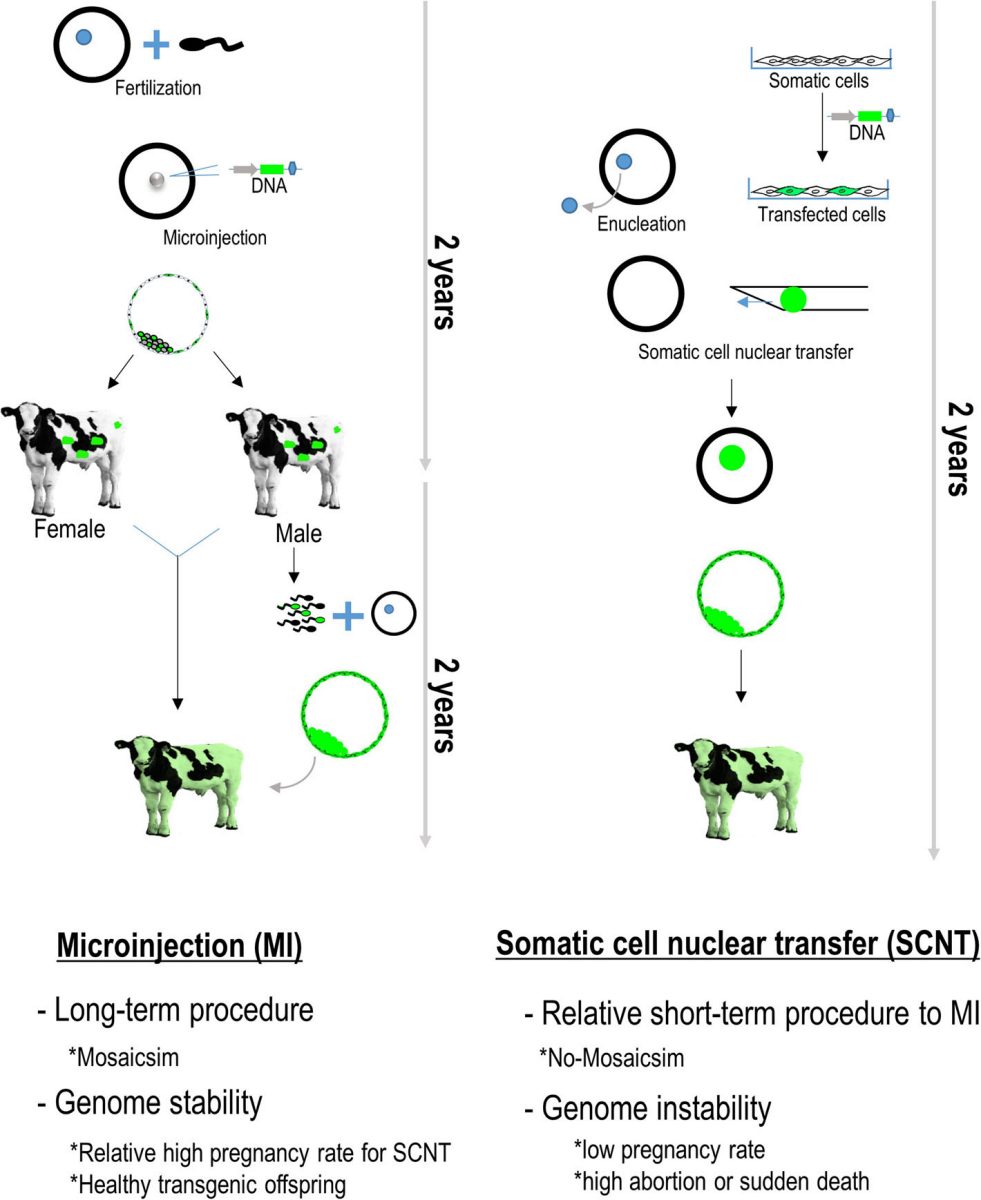
Illustration depicting micro-injection (MI) and somatic cell nuclear transfer (SCNT) for genome modified cattle (GMC). MI takes long time for GMC production without mosaicism while SCNT provides one step procedure for GMC

GMC production has progressed relatively slowly for livestock (Fig. 1) [1–3]. In the initial stage of GMC production, the plasmids including exogenous recombinant DNAs are micro-injected into in vitro fertilized embryos, similar to the procedures employed in mice. In other words, transgenic (founder) cattle are produced through the micro-injection of recombinant DNAs into the pronucleus of fertilized embryos (zygotes) and transgenesis is verified by detecting the gene [4]. Because mosaicism is observed in founder offspring, complete genetically modified mice can be produced by breeding genetically modified males or females. However, research on DNA micro-injection into bovine zygotes has progressed slowly or has been limited due to difficulties with discerning the pronucleus of fertilized embryos (Fig. 2). To observe the pronucleus of bovine zygotes, centrifugation of the denuded zygotes enables clear visualization. Bovine transgenic blastocysts produced with mechanical treatments (centrifugation and micro-injection) are transferred into the recipient cow to produce GMC. Unfortunately, the micro-injection approach is an inefficient method for production of GMC because of transgene mosaicism, low DNA delivery efficiency, long gestational periods (280 d) and puberty (around 14 mo), and single pregnancy in cattle (Fig. 3).

As an alternative to micro-injection with plasmid DNAs, high integration of a targeted foreign gene to produce GMC using a viral gene delivery system was introduced (Fig. 1) [5], and indeed, GMC have been successfully engineered via retrovirus- or lentivirus-mediated integration and have been born and grown to adults [6, 7]. However, the virus-dependent GMC approach still has limitations with regard to safety.

As an complementary procedure to micro-injection of the target DNAs or virus-infection, SCNT has been employed, in which a somatic cell, is injected into the enucleated oocytes, then fused, activated, and cultured in vitro up to blastocysts [8] (Fig. 3). Scientists think that GMC can be produced relatively easily because genome modified somatic cells can be reprogrammed into the pre-implantation stage (Fig. 3). In other words, because only genetically modified cells are selected for SCNT, there is no doubt that the pre-implantation embryos and offspring will be positive for transgenesis without mosaicism. Indeed, several transgenic cattle have been produced via SCNT [3]. However, with SCNT, the success rate of live cloned offspring is very low and abortions and abnormalities occur with a high frequency due to abnormal reprogramming [9], leading to slow progress in GMC. Nevertheless, because the method is optimal for producing complete GMC without the occurrence of mosaicism (Fig. 3), it continues to be used in the livestock field along with micro-injection.

## DNA transposons for integrating and expressing the target DNA in the bovine genome

**Fig. 4:**
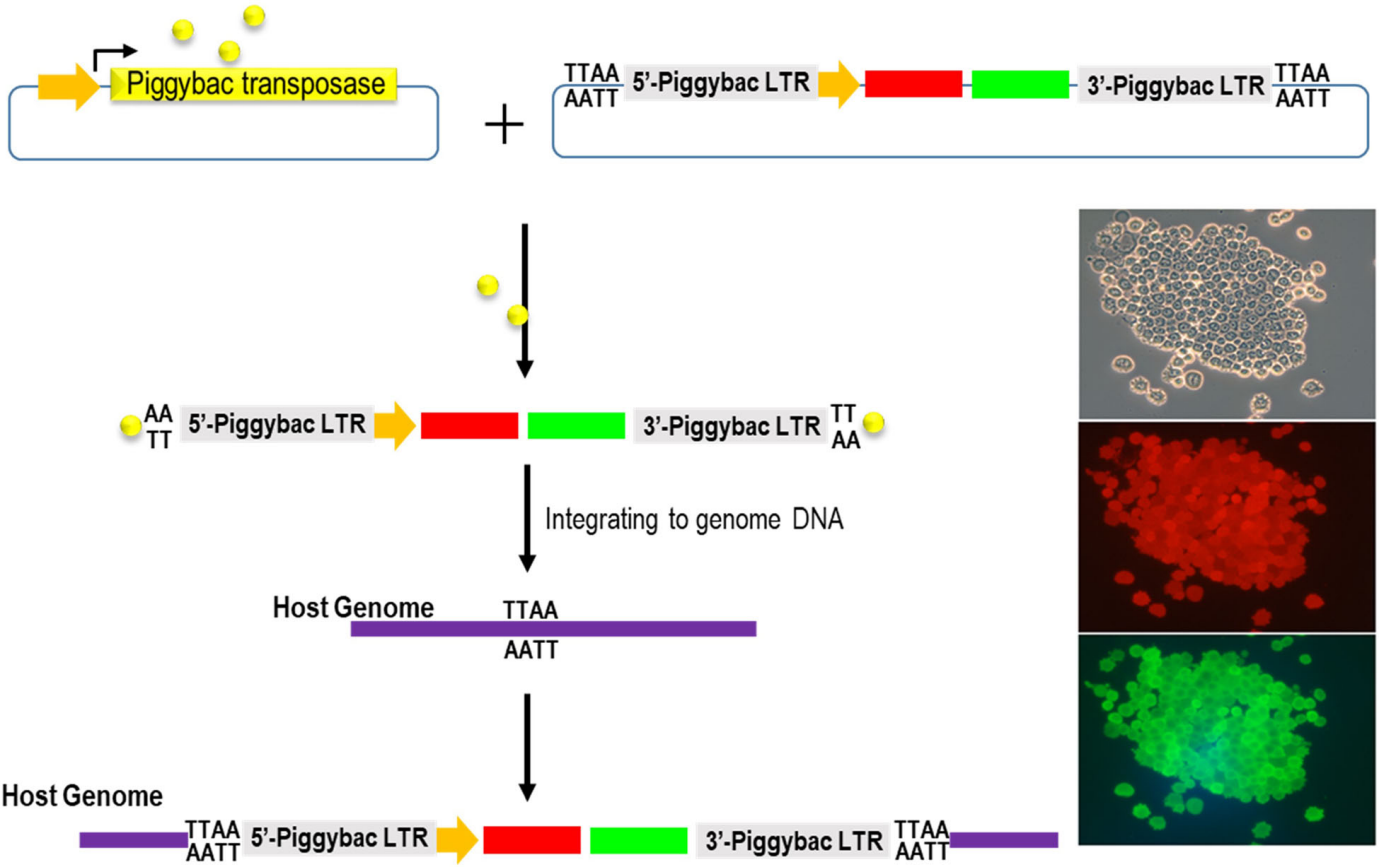
Illustration depicting genome integration via the piggyBac (PB) transposon. The PB transposase recognizes the PB-long term repeat (LTR) sequences, cuts it, and inserts itself into a “TTAA” sequence in the host genome. The inset represent Hela cells with the PB- green (G)- and red (R)-fluorescent protein (FP) gene linked by 2A peptide sequences

Due to several disadvantages (low integration efficiency, mosaicism, and mechanical stress) as previously discussed, the injection of simple plasmid DNAs into zygotes has not to be chosen for producing GMC as priority. One of the complementary options for the introduction of simple plasmid DNAs into GMC could be the DNA transposon system, because this system improves the occurrence of mosaicism and transgene integration (Fig. 4). Indeed, several GMC have been produced via DNA transposon [i.e. sleeping beauty (SB) and piggyBac (PB)] (Fig. 1) [10, 11].

The DNA transposon system is known as an efficient method for delivering foreign DNA into the host genome. Among the known transposon systems, SB and PB are primarily used for producing rodents with integrated target genes [12, 13]. The transposon delivery system has two compartments, one for transposable elements, and another for the transposase, which transpose the transposable elements into another locus of the genome (Fig. 4). Without linearization, the target gene can be more easily integrated in a specific manner using this method. SB is preferred for insertions into "TA" sites in the host genome, while PB is preferred for insertions into “TTAA” sites.

Transposon systems, where the utilization of transposon-transgenic donor cells for bovine SCNT and the production of transgenic blastocysts has been demonstrated [14, 15], integrate DNA elements into specific positions. Transposons have been integrated into the intronic region in several studies [16, 17], indicating the procedure is not harmful to cells, embryos, or animals, because it does not affect the coding region. Consistent with previous reports, we produced several transgenic cattle in our study using SB or PB [10]. In our analysis using whole genome sequencing, we encountered no issues with genome modification with regard to single nucleotide polymorphism (SNP), copy number variation (CNV), and structure variation (SV) [10], and all of the integrated DNA was founded within non-coding regions. The transgenic cattle grew up with no health issues, with the oldest being over 40 months old, and these transgenic cattle will be valuable for future studies.

**Fig. 5:**
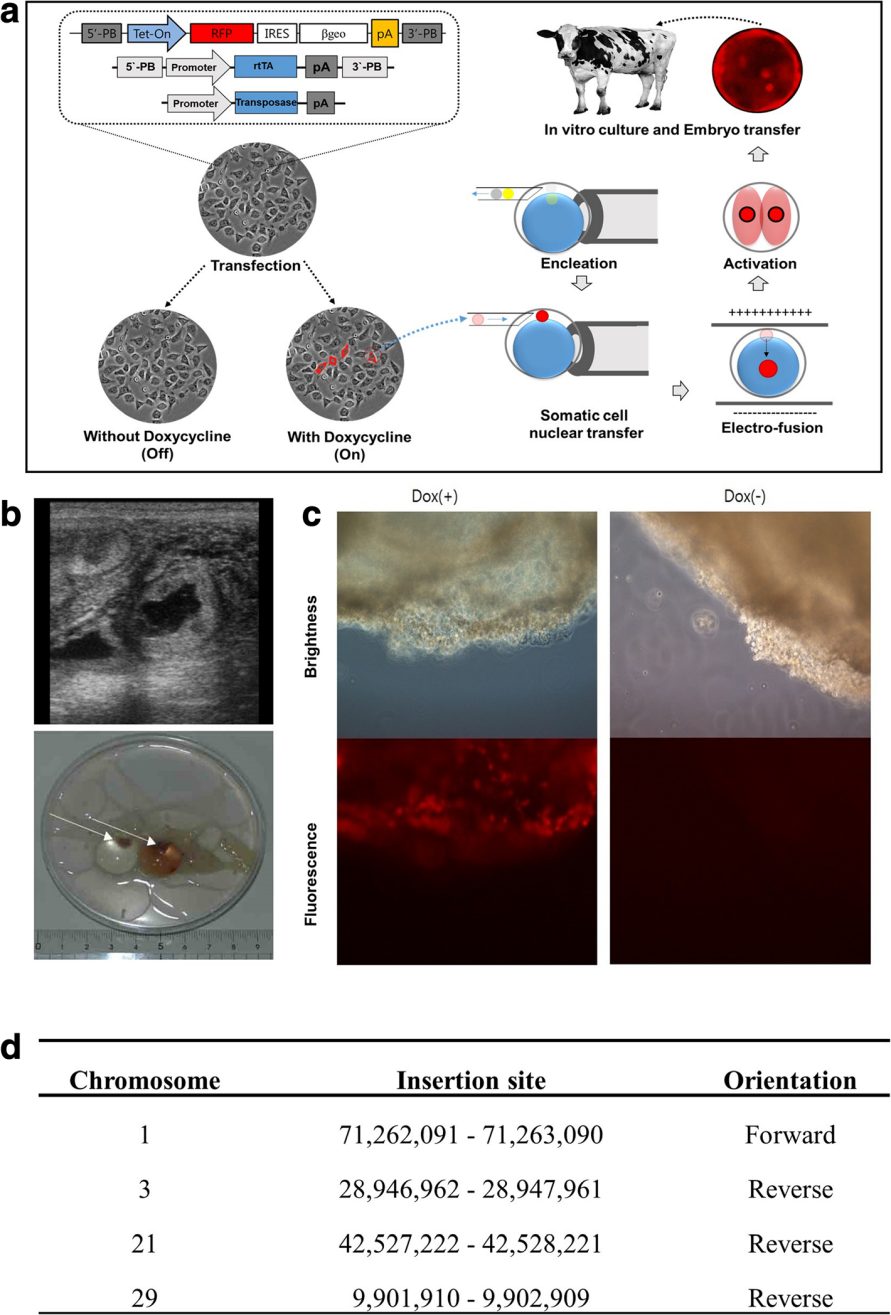
Pregnancy of cloned embryos derived from tetracycline dependent gene expression. **a** Illustration of the tetracycline dependent gene expression system in cattle; the somatic cell nuclear transfer protocol was presented in our previous publication [15]. In brief, piggyBac (PB) DNA containing red fluorescence protein (RFP) under tetracycline-controlled transcription activation promoter (tet-on) was transfected into bovine somatic cells with the PB-transposase and -reverse tetracycline-controlled transactivator (rtTA). An RFP expressing cell was microinjected into enucleated bovine oocytes, fused, and activated chemically. The blastocysts were transferred into a recipient cow. **b** Representative confirmation pictures of pregnancy using ultrasonography (upper) and collected fetuses (lower); **c** RFP expression following doxycycline treatments; to know if RFP expression was induced by tetracycline, a small piece of tissue was exposed with Doxycycline [Dox (+)] or without Doxycycline [Dox (−)]; **d** Identification of the transgene integration site via next generation sequencing analysis. Four transgene integration sites were identified

Currently, the production of transposon-based transgenic cattle utilizes ubiquitous expression of the integrated elements. In the future, tissue-specific or conditional expression [18] is needed for more precise functional analysis. Overexpression or knockout of a target gene was initially carried out, and most recent studies are focused on tissue-specific, time-dependent, or specific conditional expression in rodent models. In pig models, several conditional-gene regulated studies have been published [18–20], and additional research has been performed as well. In one report, tissue-specific GMC were produced [11], and the application of this technique is expected to increase. Another type of conditional GMC was also produced and its gene regulation was demonstrated using the Dre recombinase protein, as presented in our previous study [10]. Although the attempt at generating live tetracycline-controlled (tet-on) conditionally regulated GMC was a failure due to abnormal reprogramming, expression was confirmed in this experiment in fetal tissues (Fig. 5). In the above studies, we identified four integration sites and no genomic instabilities as well. Because all the transgene integrations were in intronic sites and no genomic instabilities were identified, we considered that failure of the cloned fetus might have been due to abnormal reprogramming. Therefore, a tissue-specific or conditional gene regulation system combined with a transposon system may prove to be a valuable tool for GMC studies, despite its’ narrow applications.

## Bovine pluripotent stem cells for GMC

The reason that research regarding genome modified mice has advanced is due to the isolation of germline transmitted embryonic stem cells, which have pluripotency. Mouse embryonic stem cells combined with homologous recombination and transgene integration have played an important role in the production of many disease or genetic mouse models [21–23]. However, in contrast to rodents, germline transmitted embryonic stem cells do not exist in livestock, though one study has reported the potential for chimerism [24]. Even though isolation of bovine embryonic stem cells from blastocysts was attempted, this endeavor failed, as after a few passages, the pluripotency of these cells disappeared [25–27]. The recent development of induced pluripotent cells, which are reprogrammed by embryonic transcription factors (Oct4, Sox2, cMyc, Klf4, and Nanog) in mice and humans [28], has raised considerable interest in researchers working with the bovine species for GMC production. Although bovine induced pluripotent stem cells have been successfully isolated and characterized [29, 30], this approach still requires more development for generating germline chimerism or long-term cultures required for genome engineering.

## Bovine genome editing for endogenous bovine genes

**Fig. 6:**
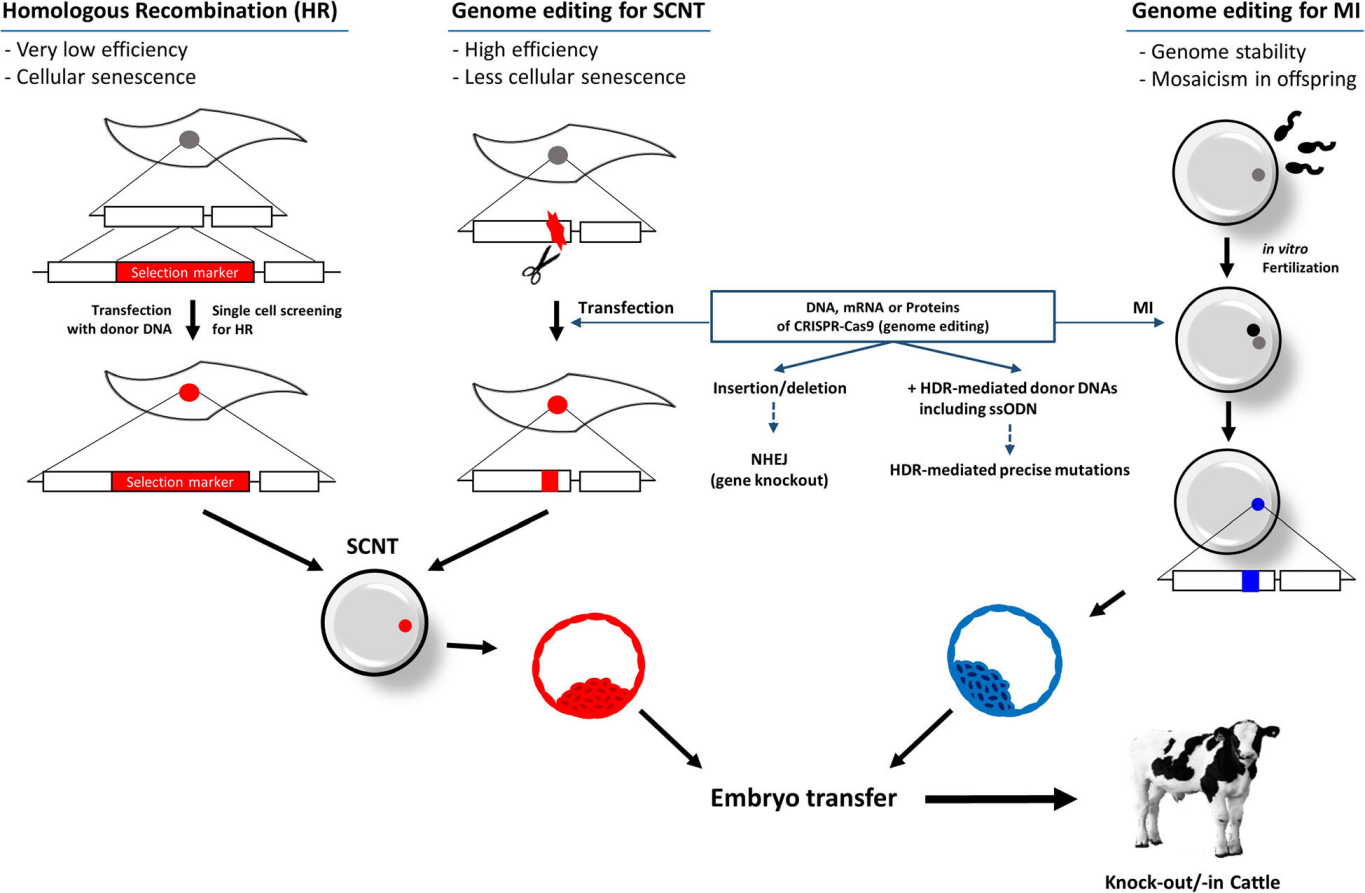
Illustration of knock-out/-in cattle. SCNT combined with homologous recombination (HR) and genome editing is a useful approach, though it is limited by abnormal reprograming and low success rates. Simple micro-injection of Cas9 and sgRNA for the target region will be useful for the production of genome edited cattle with high efficiency and genomic stability. NHEJ: Non-homologous end joining; HDR: Homology directed repair

Previously, we described how to integrate and express exogenous genes. Genome editing for endogenous genes in GMC will be reviewed in this section. Homologous recombination (HR) has been used to knockout the target region of the endogenous genome in cattle before the introduction of genome editing technologies such as Zinc Finger Nuclease (ZFN), Transcription activator-like effector nuclease (TALEN), and Clustered regularly interspaced short palindromic repeats (CRISPR)-Cas9. In mice, embryonic stem cells using HR are screened and single colony-derived cells are employed for chimerism or blastocyst complementation. However, due to the absence of embryonic stem cells in livestock, the frequency of HR events in cattle is very low. Furthermore, due to their limited life span, long-term culture of somatic cells for screening knockout-single cell colony SCNT exhibit a low efficiency in cattle (Fig. 6). As a result, since the birth of the first cloned cattle, only one knock-out/-in cattle has been born to date using SCNT combined with HR [31].

Genome editing technologies have recently been highlighted in many organisms [32]. ZFN and TALEN, which were introduced early in several fields, are being used for editing the genome in livestock. The initial adaptation of ZFN and TALEN for livestock enabled scientists to generate genome edited livestock with relatively high knockout efficiency. A few successes have been reported in cattle using ZFN [33] and TALEN [34]. Nowadays, continuous efforts to improve genome editing techniques including the use of CRISPR-Cas9 have resulted in numerous genome edited animals including cattle [3].

We believe genome editing technologies will be applied to three areas. First, the technologies will be used for basic or disease related gene function research in cattle. As previously reported, disease-related gene edited cattle have already been produced. A study reported the birth of tuberculosis-resistant cattle produced via TALEN [35]. The same procedure used for the production of virus-resistant pigs [36, 37] will also be applied in cattle for disease related studies. Studies on prion diseases using TALEN and CRISPR-Cas9 with a PRNP (prion protein) mutation enable us to produce prion-deleted cattle. In our in vitro studies, PRNP-mutated cells could be used as cell models to understand the function of the prion [38]. However, some related genes identified as candidates in mice or human cell studies are not co-related in bovine cells. Another group showed that micro-injection with Cas9 and sgRNA for PRNP may be a practical approach for future production of prion free cattle [39]. In our study, one pregnancy was identified with ultrasonography among SCNT embryos derived from PRNP mutated cells. In the future, techniques for the elimination or correction of various disease-related genes could be adapted for genome-editing in cattle.

The second area where genome editing technologies will be applied is the application of these technologies to improve genetic traits. Bovine genome sequencing revealed characteristic traits in proved bulls and traits introduced via random mutation and natural selection, such as increased muscle (myostatin gene mutation) or dehorning, were identified at the genome level. Mutated myostatin and dehorned cattle have already been born and grown into adults with the expected phenotypes (i.e., larger muscles and no horns) [34, 40]. Dehorning cattle is a low risk procedure because transgenic cattle receive dehorning genes from naturally hornless cattle. In the future, these cattle will benefit the cattle industry after the germ-line transmission is confirmed and United States Food and Drug Administration (FDA) approval is obtained for introduction into the food chain, productivity improvements, and animal welfare. Additionally, as whole genome sequencing data continue to accumulate and specific sequence variations are found [41], the combination of genome editing and genomic data will be enabled us to quickly improve genetic traits.

The third area where genome editing technologies will be applied, is in the production of designed milk or bio-pharmacological proteins can be manufactured in genome edited cattle [42, 43]. Because the cow has a very specialized system for flexible milk production, relatively simple purification and large-scale milk volume, the milk produced by cattle can be modified by genome editing of milk protein gene promoters such as by changing the protein composition or increasing some nutrients [44, 45]. Additionally, human or animal bio-pharmacological proteins can be produced on a large scale using this system. This concept of producing bio-pharmacological proteins from transgenic animals has existed for a long time and three recombinant proteins (Aytrin® from goats, Ruconest® from rabbits and Knuma® from chickens) have been approved for clinical use by the FDA. These recombinant proteins were produced via randomly mutated animals. One of the disadvantages of random mutations is that it is difficult to predict their expression levels and yields. Using genome editing with homology directed recombination, the target protein is integrated into a specific target locus with high expression (i.e., the whey acidic protein). Indeed, in a reported publication, lysostaphin was integrated into a beta-casein locus, resulting in high expression and large production volume in ZFN-treated cows [46]. Since better genome editing technologies (TALEN and CRISPR-Cas9) can now be applied to livestock, cattle with bio-pharmacological proteins can be generated.

The topic of off-target effects should be addressed in regard to genome editing technologies that generate live GMC. When a target locus was selected and designed for ZFN, TALEN, and CRISPR-Cas9, unwanted mutations have occurred at non-target loci [47–49]. Therefore, before producing GMC, DNAs, mRNA, and proteins for ZFN, TALEN, and CRISPR-Cas9, the target locus should be screened so as to select a locus with not off-target effects via in vitro assay [50].

## Public consensus on genome engineered cattle

The scientific technologies for genome editing have developed rapidly over time. However, national policies and consensus on these technologies have not caught up with current trends and there is a difference in the policies surrounding this topic in different countries. For example, SCNT-derived cattle, goats, and pigs are accepted as food in the USA (http://www.fda.gov/downloads/AnimalVeterinary/SafetyHealth/AnimalCloning/UCM124756.pdf), but not in the EU [51].

Recently, genome engineered fish (Salmon) were the first transgenic animal to receive approval as a food source in the USA and Canada [52, 53]. Additionally, several genome-edited organisms, including mushroom, have escaped from GMO regulations in the USA because they not contain any foreign DNA [54, 55]. To date, in terms of productivity, such as growth and disease resistance, genome engineered fish or plants have been approved. In the same line with livestock, productivity or disease related gene editing have been the focus of researcher, resulting in several studies, such as dehorning or double muscle, have been reported [34, 40, 56, 57]. In contrast to gene-edited plants, gene-edited animals face strict US regulation (https://www.nature.com/news/gene-edited-animals-face-us-regulatory-crackdown-1.21331). The production of gene-edited livestock is gradually increasing, and we think that it is necessary to address its’ scientific safety and efficacy. Additionally, here is also a need to promote rational regulations to guide the commercial and scientific use of GMC.

## Conclusions

Genome engineering technologies have been rapidly applied adopted for producing GMC because they have powerful advantages in the cattle industry. In the future, if policy and technological advances become harmonious, GMC will contribute to humanity and animal welfare in terms of genetic traits, disease resistance and understanding, and protein (bioreactors) production.

## Abbreviations

CNV: Copy Number Variation
CRISPR: Clustered regularly interspaced short palindromic repeats
GMC: Genome modified cattle
HR: Homologous Recombination
PB: Piggybac
SB: Sleeping Beauty
SCNT: Somatic cell nuclear transfer
SNP: Single Nucleotide Polymorphism
SV: Structure variation
TALEN: Transcription activator-like effector nuclease
ZFN: Zinc Finger nuclease
